# Langevin dynamics encapsulate the microscopic and emergent macroscopic properties of midge swarms

**DOI:** 10.1098/rsif.2017.0806

**Published:** 2018-01-03

**Authors:** A. M. Reynolds

**Affiliations:** Rothamsted Research, Harpenden, Hertfordshire AL5 2JQ, UK

**Keywords:** swarming, stochastic modelling, emergent properties

## Abstract

In contrast to bird flocks, fish schools and animal herds, midge swarms maintain cohesion but do not possess global order. High-speed imaging techniques are now revealing that these swarms have surprising properties. Here, I show that simple models found on the Langevin equation are consistent with this wealth of recent observations. The models predict correctly that large accelerations, exceeding 10 g, will be common and they predict correctly the coexistence of core condensed phases surrounded by dilute vapour phases. The models also provide new insights into the influence of environmental conditions on swarm dynamics. They predict that correlations between midges increase the strength of the effective force binding the swarm together. This may explain why such correlations are absent in laboratory swarms but present in natural swarms which contend with the wind and other disturbances. Finally, the models predict that swarms have fluid-like macroscopic mechanical properties and will slosh rather than slide back and forth after being abruptly displaced. This prediction offers a promising avenue for future experimentation that goes beyond current quasi-static testing which has revealed solid-like responses.

## 1. Introduction

Aerial mating swarms of male midges and male mosquitoes form at dawn or dusk often over prominent landmarks (hereafter called ‘swarm markers’). These swarms can contain a few hundreds, or even thousands of individuals and form to attract females from the surrounding vegetation. Females fly into these mating arenas and copulation occurs on the wing**.** In contrast with bird flocks, fish schools, animal herds and some other insect swarms (e.g. marching locusts), these cohesive swarms do not display coordinated motion. This has prompted the search for more nuanced ways to characterize collective motions in animal aggregates that go beyond the identification of global ordering or patterning.

Okubo [1] was the first to report on and attempt to characterize the three-dimensional flight patterns of swarming insects (the midge *Anarete pritchardi*). Analysis of stereoscopic photographic recordings revealed that motion inside the swarm looks more or less random in both velocity and acceleration, but each midge is, nonetheless, subject to an inward acceleration the magnitude of which increases with distance from the swarm centre. This prompted Okubo [1] to propose that midge swarms are analogous to self-gravitating systems and, as a consequence, the motion of midges within a swarm can be modelled by the Langevin equation. Ouellette and co-workers [2–8] have built on Okubo's [1] ground-breaking experiments and, in doing so, have uncovered a wealth of detailed information about the behaviours of midge (*Chironomus riparius*) swarms, resulting in the quantification of velocity and acceleration statistics, the identification of surprising macroscopic properties, including a finite Young's modulus and yield strength, and most recently reporting on the coexistence of a core condensed phase surrounded by a dilute vapour phase. In parallel with these experiments, Reynolds and co-workers [9,10] have been refining Okubo's model [1] and, in doing so, predicted that the effective attractive force towards the centre of the swarm increases both with distance from the swarm centre and with an individual's flight speed. Clear evidence of such an attractive force was subsequently found in experimental data [10]. This success suggests that generalizations of the Langevin equation may encapsulate the key dynamics of insect swarms and hence facilitate a better understanding of their collective behaviours, helping to reconcile conflicting observations made in the laboratory and in natural environments, and offering new challenges for experimentalists. Here, I show that this is indeed the case by demonstrating that the models: capture the plethora of recent observations [2–8]; predict, in accordance with observations, that correlations will be absent in laboratory swarms but present in natural swarms [3,11–13]; and predict that going beyond current quasi-static testing [4] will uncover emergent fluid-like behaviours. This is timely because the exploration of swarm ‘thermodynamics’ and the characterization of swarms in terms of state variables and constitutive laws lies at the cutting edge of swarm research [3,8]. Traditionally, models of collective motion have been validated by studying the group morphology they produce. But it is now recognized that morphology alone is not a good indicator of model correctness as different kinds of model can produce nearly identical group morphologies [7]. Models must now be able to capture both the intricate dynamics of swarms and the emergent ‘material’ properties of swarms.

## 2. Methods

### 2.1. Modelling of midge swarms

Laboratory midge (*Chironomus riparius*) swarms do not show the choreographed movements of fish schools or bird flocks, but their members do occupy just a small portion of the space available to them [2–8]. The midges appear somewhat paradoxically to be tightly bound to the swarm centre while at the same time behaving as nearly free particles inside it [7]. Here, following Okubo [1] I assume that the positions, *x*, and velocities, *u*, of such midges within laboratory swarms can be described by the stochastic differential equations
2.1
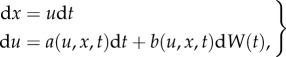
where d*w*(*t*) is an incremental Wiener process with correlation property 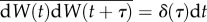. Equation (2.1) is effectively a first-order autoregressive stochastic process in which position and velocity are modelled as a joint Markovian process. At second order, position, velocity and acceleration would be modelled collectively as a Markovian process. Physically, this hierarchy of models corresponds to the inclusion of a velocity autocorrelation timescale, at first order, and to the addition of an acceleration autocorrelation timescale, at second order, and so on [14]. Here, the deterministic term, *a*(*u*, *x*, *t*), is determined by the requirement that the statistical properties of the simulated trajectories be consistent with the observations of Kelley & Ouellette [2]. Kelley & Ouellette [2] showed that: (i) the spatial distribution individuals from the swarm centre is approximately Gaussian in all three dimensions and weakly axisymmetric; (ii) and that, in sufficiently large swarms, individual velocity distributions have long, nearly exponential tails.

Mathematically, these consistency conditions require that the joint distribution of velocity and position *P*(*u*, *x*, *t*) be a solution of the Fokker–Planck equation [15]
2.2
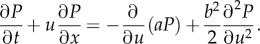


Here, in broad agreement with the observations of Kelley & Ouellette [2], I assume that positions and velocities can be separated and are distributed according to
2.3
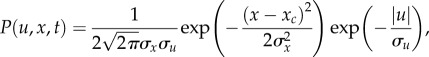
where *x_c_* is the location of the swarm centre, *σ_x_* is the root-mean-square position and *σ_u_* is the root-mean-square speed. Equation (2.2) implies that

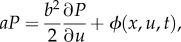
where, for statistically stationary swarms having *∂P*/*∂t* = 0,
2.4
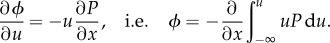


It follows from equations (2.2), (2.3) and (2.4) that
2.5
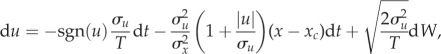
when, without loss of generality, 
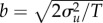
, where *T* is a velocity autocorrelation timescale. The first term is a ‘memory term’ which causes velocity fluctuations to relax to their mean value. The second term is the ‘conditional mean acceleration’, and the third term is the stochastic driving noise. In accordance with observations, the mean acceleration increases linearly with distance from the swarm centre and with speed relative to the swarm centre [2,10]. Note, however, that in small swarms (less than 10 individuals) velocities are observed to be Gaussian rather than exponentially distributed [1,2], and in this case a directly analogous calculation gives
2.6
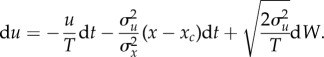
This model is identical to the model posited by Okubo [1]. The linear increases in mean acceleration (force) with distance from the swarm centre in the two models, equations (2.5) and (2.6), are consistent with midge swarms behaving as self-gravitating systems [1]. This is consistent with midges interacting primarily via long-range acoustic stimuli and with ‘adapting’ their response to the overall sound level so that acoustic sensitivity drops when there is a strong background noise [16]. Adaptivity is a common feature of biological sensory systems. Gorbonos & Gov [17] showed that adaptivity also prevents collapse of the swarm and therefore confers on the swarm a natural stability mechanism. This is related to Jeans instability, which in stellar physics causes the collapse of interstellar gas clouds and hence star formation when the internal gas pressure cannot prevent gravitational implosion. Two- and three-dimensional models can be formulated in a directly analogous way [10] but, in contrast to one-dimensional models, they are not uniquely determined by prescribed distributions of position and velocity. Models differ in the propensity to which simulated trajectories tend to orbit around the swarm centre. Models of midge swarms producing orbiting trajectories can currently be discounted because there are no reported observations of such behaviours.

In contrast to laboratory swarms, the velocities of midges within natural swarms are correlated [11,12], i.e. the midges are effectively interacting by velocity matching. Later I suggest that the correlations are induced by environmental disturbances. The modelling framework can be extended to take explicit account of such interactions between individuals, following the approach of Thomson [18], who devised a stochastic model for the motion of particle pairs in turbulence. One of the simplest such models is given by
2.7
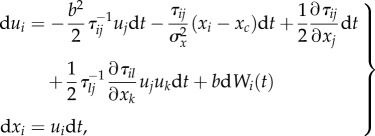
where the subscripts denote different individuals, *τ* is the velocity correlation matrix with components 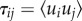, 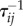 denotes components of *τ*^−1^ and where d*W*(*t*) is an incremental Wiener process with correlation property 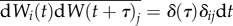. Modelled velocities are Gaussian with mean zero (and close neighbours will have similar velocities by virtue of the correlations). The first term describes how an individual velocity relaxes to a weighted sum of the velocities of its neighbours. The second term is an attractive force that binds individuals to the swarm centre. The third and fourth terms ensure that the spatial distribution of individuals is uniform on average. Without these terms, individuals would tend to drift apart because relative velocities tend to decrease as individuals come together and increase as they move apart, leading to a net outward drift; a process akin to turbophoresis. The third and fourth terms counter this drift which, on average, is given by 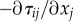. They are, in effect, a velocity-dependent mean acceleration. The model, equation (2.7), reduces to the phenomenological model of midge swarms proposed by Passino [19] when the fourth term is averaged over velocity, i.e. when 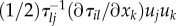 is approximated by 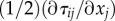. Passino [19] noted that such a term represents a short-range ‘repulsive’ interaction when velocity correlations are positive. When a pair of simulated midges are much closer than the correlation length scale, the acceleration between them becomes strongly repelling. This is consistent with the observations of Puckett *et al*. [7] who reported that accelerations become strongly repelling when the separation between a pair of midges is less than 12 mm (about two body lengths), a separation comparable to the correlation length scale, 26 mm [3].

## 3. Results

### 3.1. Ramifications of speed-dependent effective forces: biological insights

Reynolds & Ouellette [9] recognized that Lévy flight patterns can result from speed-dependent effective forces and they found some support for such flight patterns in laboratory swarms of the midge *Chironomus riparius.* The occurrence of Lévy flight patterns may be accidental but they may have biological significance. Lévy flight patterns could provide males with a highly effective searching strategy for locating females that have flown into the swarm [9]. This may be biologically significant because competition within the swarm appears to be a scramble to be the first to locate a female which may actively attempt to avoid capture [20]. The identification of speed-dependent effective forces may also lead to a more detailed understanding of the origins of interactions between midges which probably arise from acoustic sensing [10].

### 3.2. Ramifications of speed-dependent effective forces: large accelerations

**Figure 1.**
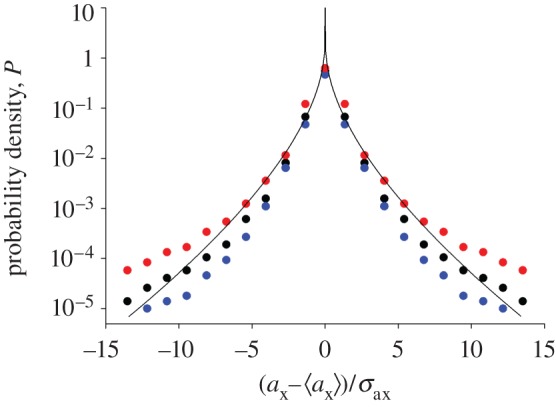
Individual midge accelerations. Standardized acceleration probability density functions. Model predictions for speed-dependent forces are shown (solid line) together with observations (black filled circle, median recording; red filled circle, maximum recording; blue filled circle, minimum recording) of horizontal accelerations [2]. There are no adjustable parameters in the predicted standardized pdf. Models with speed-independent forces predict that accelerations are Gaussian distributed.

Another ramification of speed-dependent forces, the occurrence of very heavy tailed distributions of unconditional accelerations (resulting in accelerations exceeding 10 g), has been hiding in plain sight [1,2]. The distribution of unconditional accelerations is determined by
3.1
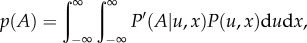
where 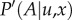 is the conditional distribution of accelerations. It is seemingly natural to suppose that conditional accelerations are Gaussian distributed with mean 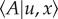 and variance 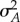. Mean accelerations are observed to increase linearly with distance from the swarm centre, and swarm profiles are, to good approximation, Gaussian [2]. It follows from these observations and from equation (3.1) that the distribution of unconditional accelerations will also be Gaussian if mean accelerations are independent of velocity. Such distributions of acceleration are not observed. Heavy-tailed distributions of unconditional accelerations can only arise when the mean accelerations depend on both position and speed. For example, for the model given in equation (2.5), evaluation of equation (3.1) in the saddle point approximation gives a stretched exponential distribution,
3.2
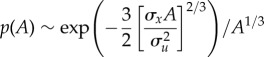
(‘∼’ means distributed as). This prediction compares favourably with the observations of Kelley & Ouellette [2] (figure 1). This shows that for the observed distributions of velocity and observed speed–position-dependent mean accelerations to be consistent with the observed unconditional distribution of acceleration, the distribution of conditional accelerations must (to good approximation) be Gaussian. In other words, the heavy tails of the unconditional distribution of acceleration are not indicative of new dynamics beyond those characterized by the velocity distribution and the mean acceleration statistics. This finding mirrors that of tracer-particle accelerations in high Reynolds-number turbulence which also have stretched-exponential distributions [21] and which have been interpreted within the context of ‘superstatistics’ where one has a superposition of Gaussians whose variance fluctuates over a wide spatial–temporal range [22,23].

### 3.3. Emergence of fluid-like macroscopic mechanical properties

**Figure 2.**
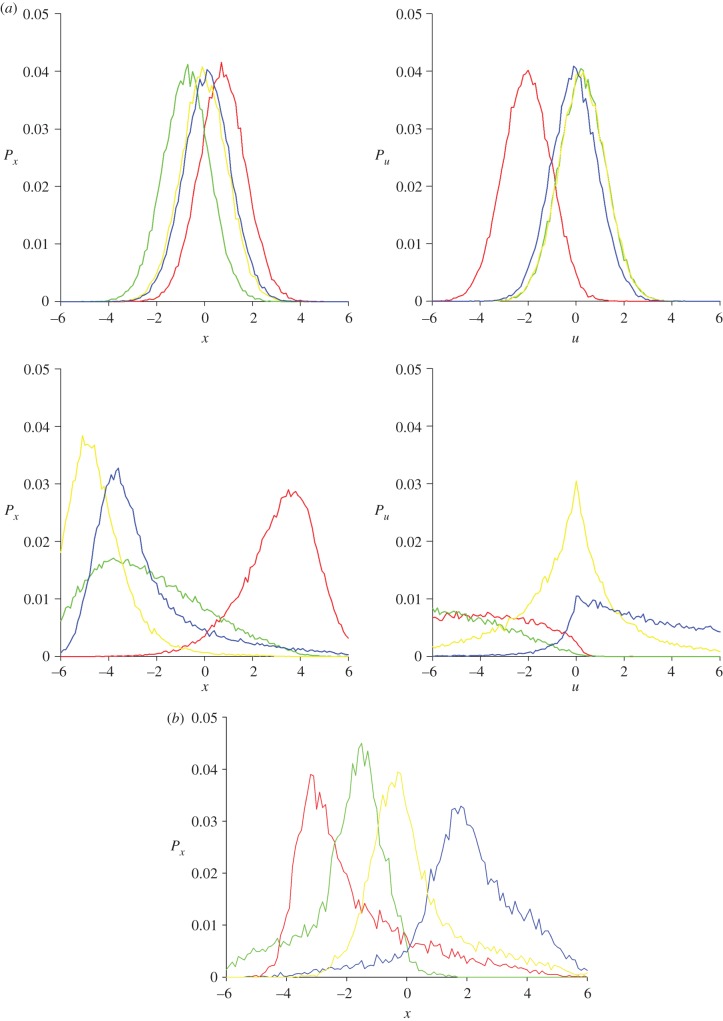
(*a*) Predicted emergence of solid-like and fluid-like behaviours. Distributions of positions, *P_x_*, and velocities, *P_u_*, at times *t* = 2T (red lines), *t* = 4T (green lines), *t* = 6T (yellow lines) and *t* = 8T (blue lines) after at swarm at equilibrium and centred on *x* = 0 is suddenly displaced to *x* = 5. Predictions for speed-dependent forces (lower panel) and speed-independent forces were obtained using equations (2.5) and (2.6). A fluid-like ‘sloshing’ behaviour is predicted to arise when forces are speed-dependent, and a solid-like ‘sliding’ behaviour is predicted to arise when forces are speed-independent. Predictions were obtained for *σ_x_* = 1, *σ_u_* = 1 and *T* = 1 a.u. (*b*) Predicted emergence of solid-like and fluid-like behaviours. Distributions of positions, *P_x_*, and velocities, *P_u_*, at times *t* = 2T (red lines), *t* = 4T (green lines), *t* = 6T (yellow lines) and *t* = 8T (blue lines) after a swarm at equilibrium and centred on *x* = 0 is suddenly displaced to *x* = 5. A fluid-like ‘sloshing’ behaviour is predicted to occur as the swarm returns to equilibrium. Predictions were obtained using equation (2.7) for a swarm containing 10 midges with a Gaussian density profile and exponential velocity correlations, 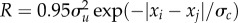, and characterized by *σ_x_* = 1, *σ_u_* = 1, *σ_c_* = 1 and *b* = 1 a.u. Fluid-like behaviours are also predicted to emerge when correlations are weaker, i.e. when 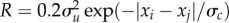, and when they are shorter-ranged, i.e. when *σ_c_* = 1/2.

The coordinated movements of individuals within strongly correlated aggregations such as bird flocks is visually striking and, as a result, much research has been directed at characterizing the conditions under which ordering will emerge. But insect swarms can behave collectively without ever exhibiting long-scale order, prompting the search for different descriptions. Ni *et al*. [4] have suggested that the dynamics of insect swarms may be characterized in terms of macroscopic state variables and constitutive laws instead of low-level interactions. Ni & Ouellette [3] subsequently showed that single laboratory swarms can be pulled apart into multiple daughter swarms and that when this done quasi-statistically, swarms appear to be more solid-like rather than liquid- or gas-like, in that they have a finite Young's modulus and yield strength but do not flow like viscous fluids. Nonetheless, model predictions suggest that fluid-like properties will emerge in dynamic tests as a consequence of speed-dependent restoring forces. Simulations with speed-independent forces (equation (2.6)) predict that after being suddenly displaced from its marker, a swarm will eventually return to its equilibrium position after ‘sliding’ back and forth past the marker while maintaining its equilibrium density profile (figure 2*a*). Simulations with speed-dependent forces (equation (2.5)), on the other hand, predict a fluid-like response, as the swarm ‘sloshes’ back and forth past the marker before returning to equilibrium (figure 2*a*). These predictions do, however, presuppose that the non-equilibrium dynamics do not differ from the equilibrium dynamics as encoded in the model and which are derived from equilibrium statistics. The distinction between speed-independent and speed-dependent forces is, however, predicted to be of importance only after relatively large, sudden perturbations and will not manifest itself in quasi-stationary tests. Natural swarms are also predicted to have fluid-like properties because accounting for correlations leads to speed-dependent restorative forces (equation (2.7)) (figure 2*b*). If correlations were not accompanied by speed-dependent restorative forces, then they might be expected to endow swarms with solid-like macroscopic properties.

### 3.4. Phase coexistence

**Figure 3.**
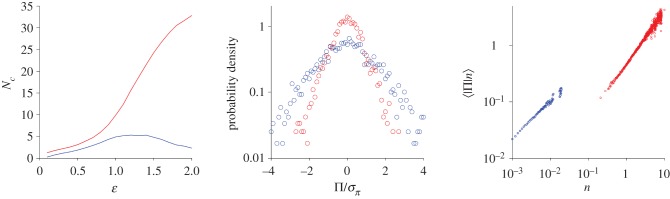
Topological analysis of the simulated swarm structure and pressure statistics. A three-dimensional spherically symmetric swarm containing 38 individuals was simulated using the model of Reynolds *et al*. [10] with speed-independent forces. The topological structure of the simulated swarm was identified by creating a simplicial complex from the positional data by associating each one with a sphere of radius *ɛ*/2. The simplicial complexes were then quantified in terms of the number, *N_c_*, of connected components in the complex. When *ɛ* is very small, all insects will appear to be isolated, and, conversely, when *ɛ* is very large, all insects will be part of the same connected component. Here, a single large cluster (which lies in the middle of the swarm) (red line) is seen to be surrounded by small unconnected components (blue line). The ‘condensed core’ and ‘dilute vapour phases’ are seen to have distinct pressure statistics. Here, pressure 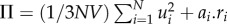 where *V* is the volume of the phase, *N* is the number of constituent individuals, *u_i_* is the velocity of insect i, *a_i_* is its acceleration and *r_i_* is its distance from the swarm centre. Predictions do not change significantly when forces are taken to be speed-dependent. Model predictions are strikingly similar to the observations of Sinhuber & Ouellette [8].

Midges in laboratory swarms are very weakly correlated, with correlation lengths of only a few body lengths. These swarms are, nonetheless, behaving collectively. A recent study showed that laboratory swarms consist of a core ‘condensed’ phase surrounded by a dilute ‘vapour’ phase [8]. These two phases maintain distinct macroscopic properties even though individual insects pass freely between them. The emergence of such phases is predicted by three-dimensional models of uncorrelated swarms with and without speed-dependent forces [10] (figure 3). In this modelling, midges are collisionless and their dynamics are not governed by interactions between midges, but instead are governed by the overall forces which binds the swarm to its centre. In accordance with observations [8], the model predicts that (at least approximately) the average pressure of the condensed phase increases linearly with density so that 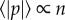 (i.e. it's thermal). The average pressure of the vapour phase is observed and predicted to increase sublinearly with density so that 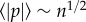. From a thermodynamic perspective this is strange because the scaling exponent, *γ*, is expected to be the ratio of heat capacities, *c*_p_/*c*_v_ and thus greater than unity because, at constant pressure, specific heat is always greater than that at constant volume. Moreover, for an ideal gas *γ* = 1 + 2/*N* where *N* is number of the degrees of freedom of a molecule.

### 3.5. Velocity correlations in laboratory and natural swarms

**Figure 4.**
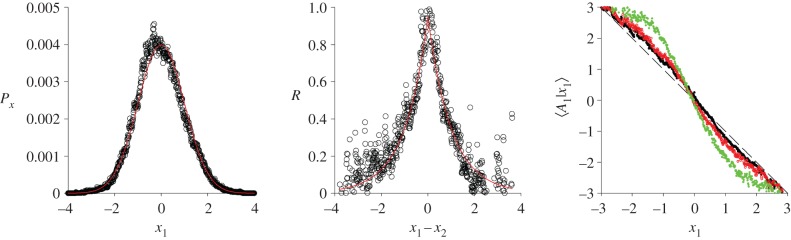
Predicted enhancement of attraction to the swarm centre due to correlations. Predictions were obtained using the model, equation (2.7), for correlated swarms. Model outputs (o) for the swarm density profile, *P_x_*, and the velocity correlations, *R*, are seen to be consistent with model inputs (red solid lines), i.e. with a Gaussian density profile and exponential velocity correlations, 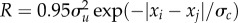. This demonstrates that the model is working correctly. Mean accelerations (forces) towards the swarm centre are greater than they are in uncorrelated swarms (black dashed line). Predictions are shown for a swarm with *N* = 3 insects having *σ_x_* = 1, *σ_u_* = 1, *σ_c_* = 1 and *b* = 1. This enhancement with *N* (*N* = 5, red open circle; *N* = 10, green open circle).

**Figure 5.**
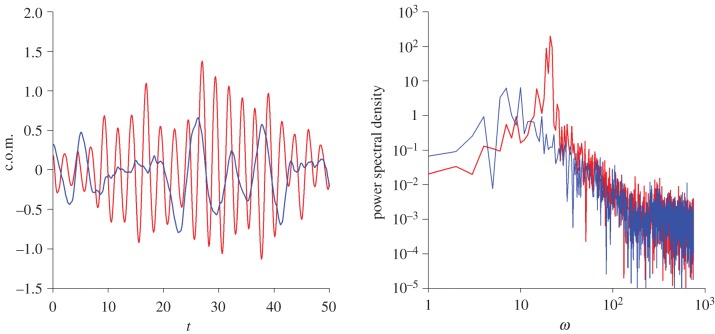
Correlations are predicted to result in coherent periodic motion (dancing). Predictions of the motion of a swarm's centre of mass obtained using the model, equation (2.7), for correlated swarms (red lines). Predictions are shown for a swarm with *N* = 10 insects having exponential correlation and *σ_x_* = 1, *σ_u_* = 1, *σ_c_* = 1 and *b* = 1. Shown for comparison are model predictions for an uncorrelated swarm with *N* = 10, *σ_x_* = 1, *σ_u_* = 1, *σ_c_* = 0 and *b* = 1 a.u. The spike in the spectrum is indicative of near-periodic motion.

Natural swarms of the midges *Cladotanytarsus atridorsum*, Chironomidae and Ceratopogonidae, and of the mosquitoes *Anopheles gambiae and Anopheles coluzzii* display strong correlations that are totally incompatible with models of non-interacting midges [11–13]. The correlation scale increases with the swarm size, and this was interpreted by Attansasi *et al*. [12] under the guise of criticality. This is markedly different from laboratory swarms which are uncorrelated. Ni & Ouellette [3] suggested that a likely explanation for this difference is the influence of external environmental factors. Laboratory swarms are very well controlled, with no temperature gradients, air flows or other dynamic disturbances. In natural swarms, all of these influences are unavoidably present. The onset of correlated movements may therefore be triggered by the presence of external perturbations, as seems to happen in laboratory swarms whose centre of mass traces out elliptical, oscillatory trajectories when the swarm is disturbed by periodically modulated sound recordings of male midges [5]. This possibility finds support in the results of numerical simulations for correlated swarms obtained using equation (2.7) (figure 4). Correlations are seen to enhance the strength of the effective force that binds the swarm to its centre, and this effect is seen to be maximal when the correlation scale is comparable with the swarm size. This can also be seen analytically by integrating the conditional mean acceleration (equation (2.7)) for one individual, over the positions and velocities of all other individuals. Correlations may therefore help to maintain the coherence of the swarm and hence be selected for, as swarm coherence may promote the collective signalling to females [13]. In accordance with observations, correlations are also predicted to result in coherent ‘dancing’ of the swarm [11], and ‘milling’ of the centre of mass [5] (figure 5). The ‘dancing’ seen in natural swarms [11] may, in fact, be ‘sloshing’ because such swarms are predicted to have fluid-like properties. Correlations are also predicted to enhance the dispersal (normal diffusion) of swarm centroids after swarms break free from their nucleation markers (results not shown).

Conversely, anti-correlations (episodes of antiparallel flight) reduce the strength of the effective force that binds swarms to their centres. Puckett *et al*. [24] reported that midges spend about 15% of their time engaged in nearly harmonic oscillations conducted in synchrony with other midges, and that these pairwise interactions do not typically occur between midges that are nearest neighbours. The predicted incumbent weakening of the effective binding force may be an accidental (mathematical) consequence of these pairwise interactions, which nonetheless could be crucial if laboratory swarms are to break free from their markers and hence crucial for the emergence of true swarming behaviour. In any case, the weakening due to anti-correlations cannot be as pronounced as the strengthening due to correlations, as any number of midges can be correlated but only pairs of midges can be maximally anti-correlated. Anti-correlations have, in fact, only been observed between pairs of individuals in laboratory swarms [24], whereas correlations (parallel flights) are present in pairs and within larger subgroups [11–13].

### 3.6. Ground effects

**Figure 6.**
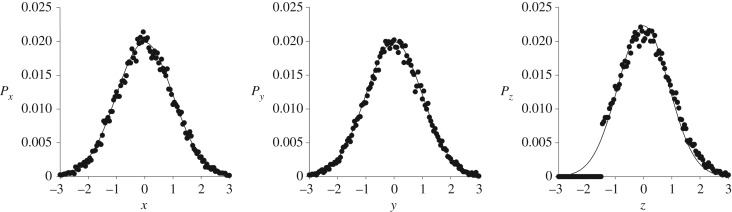
Predicted distortion in the vertical swarm density profile due to the presence of the ground. A spherical symmetric swarm centred on *x* = *y = z* = 0 was simulated using the model of Reynolds *et al*. [10]. A reflective boundary condition was imposed at height *z* =−1.5 to mimic the expected influence of the ground. Model predictions (o) are shown together with unperturbed Gaussian density profiles (solid-lines) with equivalent means and variances. In accordance with observations [2,4], above the swarm there are diffuse trajectories, as individuals entering or leaving the swarm tend to do so from above.

In contrast with wild swarms and bird flocks, laboratory swarms are only weakly axisymmetric. Large swarms are longest in the vertical dimension, whereas small swarms are elongated in a horizontal direction [2]. Kelley & Ouellette [2] speculated that this might be because individuals join the swarm by flying in from above, thereby extending the large swarm in the vertical direction. This was later corroborated by Ni & Ouellette [4] who reported that above the swarm there are diffuse trajectories, as individuals entering and leaving the swarm tend to do so from above. The results of numerical simulations (figure 6) suggest that this is not a novel dynamic, different from the swarm dynamics encoded in the model, but may just be a consequence of the ground inhibiting downward movement of the swarm. In the modelling, such an impermeable barrier to movement was implemented through the imposition of a simple reflective boundary condition which is seen to distort the swarm profile in the vertical direction but not in the horizontal directions (figure 6). At the boundary, the vertical component of velocity changes sign. Further analysis is presented in appendix A.

## 4. Discussion

Midge swarms behave collectively without displaying coordinated motion of the kind seen in bird flocks, animal herds and fish schools [2], and in the laboratory are only weakly correlated [5]. This has prompted the search for more general indicators of the collective nature of swarming. In a pioneering suite of studies, Ouellette and co-workers [2–8] have revealed that insect swarms have surprising macroscopic mechanical properties that can be characterized in terms of macroscopic state variables and by constitutive laws instead of low-level interactions. Here, I showed that many of these surprising properties along with other facets of insect swarms are predicted by simple models based on the Langevin equation; models that are close relatives of Okubo's [1] original model of midge swarms.

The models are easy to formulate, computationally inexpensive and can be studied analytically. They were shown to predict correctly many recent and intriguing observations including: the occurrence of anomalously large accelerations [2]; the presence of a condensed core surrounded by a dilute vapour that does not have a straightforward thermodynamic counterpart [8]; and emergent macroscopic mechanical solid-like properties [4]. The models also predict the occurrence of fluid-like behaviours which await experimental verification and thereby provide new lines of enquiry beyond the static [8] and quasi-static tests [4] which have revealed emergent macroscopic mechanical properties similar to solids that do not flow like viscous fluids.

The models may also help to reconcile seemingly conflicting reports about the importance of correlations, hinting at the potential importance of environmental conditions on collective behaviours [11–13]; and conversely, provide new insights into the practical implications of the occurrence of anti-correlated pairs in laboratory swarms. It has been hypothesized that correlations (i.e. parallel flights) allow for mate recognition via wingbeat frequency matching [25], and that observed interactions represent a means of obtaining information on what may be occurring in a part of the swarm outside an individual's perceptive range [13,26]. It has even been suggested that the males are competing for space with the swarm, so that the parallel flights are a form of ritualized aggression in males [27]. But none of these hypotheses are consistent with the presence of correlations of natural swarms, and their absence (or replacement by anti-correlations) in laboratory swarms [3,24]. Here it was suggested that by increasing the strength of the effective force that binds midges to the swarm centre, correlations help natural swarms to counter disturbances due to environmental perturbations; and conversely, that anti-correlations might help laboratory swarms to break free from their swarm markers. This hypothesis could be tested in the laboratory.

## Data accessibility

Computer codes can be obtained from the author.

## Appendix A

**A.1. Stochastic modelling of the swarms' exterior**

Mean accelerations (restoring forces) are observed to increase linearly with distance from the swarm centre [1,2], and hence midge swarms are behaving like self-gravitating systems [1]. This is not unexpected as midges are thought to interact primarily via long-range acoustic sensing and because acoustic and gravitational sources decay in a similar way [16]. Outside of the swarm, mean accelerations are therefore to be expected to decrease linearly with the square of the distance from the swarm centre [28]. Okubo's [1] model for within-swarm trajectories, equation (2.6),  can therefore be supplemented by an additional model

A 1

for outside-swarm trajectories. Within-swarm positions and velocities remain Gaussian. Outside the swarm, velocities are also Gaussian, but the background equilibrium distribution of midges is given by

A 2

If , then the density profile is continuous across the edge of the swarm, and individuals can move freely in and out of the swarm, as is observed to occur at the top of large swarms [2,4]. Other choices for the far field midge concentration, *ρ*_0_, result in discontinuous density profiles and confinement as mean accelerations are determined by  (see equation (2.4)). The extension to non-Gaussian velocity statistics is straightforward and as before leads to velocity-dependent mean accelerations that have similitude with Gerber's [29] long-forgotten controversial theory of speed-dependent gravity. Intriguingly, by reconciling an inverse-square restoring force with homogeneous velocity statistics, the new model, equation (A 1), has resonance with modified Newtonian dynamics [30], a contemporary but controversial theory of gravity. Modified Newtonian dynamics may therefore govern the transfer of midges into and out of the swarm, while Gerber's gravity governs what happens inside the swarm.
